# Breastfeeding Support and Protection During Natural Disaster and Climate-Related Emergencies in Indonesia: Policy Audit

**DOI:** 10.1177/08903344251322770

**Published:** 2025-03-20

**Authors:** Andini Pramono, Alvia Hikmawati, Setiya Hartiningtiyaswati, Julie Smith

**Affiliations:** 1 National Centre for Epidemiology and Population Health, Australian National University, Canberra, ACT, Australia; 2 Indonesian Breastfeeding Mothers Association, Jakarta, Indonesia; 3 Surabaya Institute of Health and Business, Surabaya, Indonesia

**Keywords:** breastfeeding, breastfeeding support, Global Strategy for Infant and Young Child Feeding, policy analysis, public health

## Abstract

**Background:**

Indonesia is a middle-income country in Southeast Asia in which 2,394 disasters were recorded in 2022 alone, with a total loss of 178,367 lives. In 2018 governments at the World Health Assembly resolved to improve emergency planning using Operational Guidance on Infant and Young Child Feeding in Emergencies (OG IFE). Little is known about whether Indonesian policies protect the health and lives of women, infants, and young children in line with OG IFE through planning for breastfeeding support and protection during emergencies.

**Research Aim:**

To identify and audit Indonesian policy regulations regarding infant and young child feeding support and protection during emergencies.

**Method:**

A search of the grey literature was conducted in 2023 through Google Basic and Advanced Search, official websites, and consultation with the Indonesian Breastfeeding Mothers Association (Asosiasi Ibu Menyusui Indonesia/AIMI) network. Keywords used included the Indonesian words for “regulation,” “disaster,” and the name of a province, city, or region, or the name of appropriate government organizations. Data was analyzed using a qualitative content analysis approach, and based on the Infant and Young Child Feeding in Emergencies: Operational Guidance for Emergency Relief Staff and Program Managers (OG-IFE) framework.

**Results:**

A total of 513 regulations were found online; however, only four were included for audit. The 509 excluded regulations did not specifically mention infant and young child feeding in emergencies. Those Indonesian policies that did mention infant and young child feeding in emergencies lacked attention to, and comprehensive planning for, breastfeeding protection and support during emergencies.

**Conclusion:**

Mothers and infants may be exposed to unnecessary risk and avoidable morbidity and mortality during emergencies. Indonesia could enhance its disaster relief policies to align with international standards supporting breastfeeding in emergencies. This could involve more comprehensive and integrated regional planning and preparation before disasters, including regular assessment of local infant and young child feeding practices, communication measures to raise emergency workers' awareness of breastfeeding, and resourcing of training so as to translate policies into practice.

## Key Messages

- Indonesia is prone to national and localized disasters and has committed to better planning for optimal infant feeding inemergencies based on the relevant operational guidance (OGE-IFE) since 2017. Progress on this in Indonesia was unknown.
- In 2023, only four government regulations in Indonesia addressed optimal infant feeding in emergencies, all at a lower government level and related to specific regions or disasters.
- Just three regulations covered the critical issue of controlling breast milk substitute donation and distribution in emergencies, and these may not be effective in practice due to gaps in resourcing and lack of measures to increase emergency worker awareness and training.
- Together with international and local breastfeeding support organizations, Indonesia should develop its planning for infant and young child feeding in emergencies to address major planning and practice gaps. These should be in line with international national guidance and ensure resources are present that incorporate OG-IYCF-E guidelines into both policy and practice at all levels of government in this disaster-prone country

## Background

Climate change has been identified as a global health threat. The World Health Organization (WHO) stated that climate change is associated with an additional 250,000 deaths every year from diseases like malaria, diarrhea, and heat exhaustion (WHO, 2021). In the last 50 years, the number of natural disasters has increased fivefold due to climate change and rising temperatures (United Nations, 2023). It is estimated that the earth’s temperature will increase by around 1.1–1.8 degrees Celsius between 2023 and 2027 (World Meteorological Organization, 2023). Rising air temperatures will cause sea levels to rise, storms and wind speeds to intensify, drought and wildfire seasons to intensify and lengthen, and precipitation and flooding to increase (Oxfam International, 2023). Over half (51.49%) of climate-related deaths are due to non-optimal temperatures occurring in Asia (Zhao et al., 2021). Unfortunately, the people affected the most are those who are least able to protect themselves and their families, including infants and young children (WHO, 2023).

In 2018 governments at the World Health Assembly (WHA) resolved to improve emergency planning for infant and young child feeding. Recognizing that appropriate, evidence-based, and timely support of infant and young child feeding in emergencies saves lives, protects child nutrition, health and development, and benefits mothers and families; Resolution WHA 71.9 urged all Member States to “take all necessary measures to ensure evidence-based and appropriate infant and young child feeding during emergencies, including through preparedness plans, capacity-building of personnel working in emergency situations, and coordination of intersectoral operations.” (page 2) The WHO was also asked to develop tools for training, monitoring, advocacy, and preparedness for the implementation of the operational guidance on infant and young child feeding in emergencies and support Member States to review experiences in its adaptation, implementation, and monitoring.

Breastfeeding saves lives and protects the health of both mothers and infants in ordinary times and in disasters and emergencies. For example, every year, at least 595,379 childhood deaths (from ages 6–59 months) from diarrhea and pneumonia could be prevented by breastfeeding (Walters et al., 2019). In the long term, breastfeeding also benefits the country by bringing many public health benefits (Binns et al., 2016). At the global level, breastfeeding contributes to climate protection because it produces a far lower carbon footprint compared to feeding with commercial milk formula products (Andresen et al., 2022; Karlsson et al., 2019). Human milk is 100% sustainable, uses few of the planet's increasingly scarce land and water resources, emits no greenhouse gases, and produces less waste than feeding with commercial milk formula products (Dadhich et al., 2021). Climate change mitigation from protecting breastfeeding in Low and Middle-Income Countries (LMIC) is a co-benefit of better enabling breastfeeding, and this “carbon offset” effect can now be measured using the Green Feeding Tool (Smith et al., 2023, 2024), which is a free online tool to estimate carbon and water footprints related to commercial milk formula use among infants under 6 months (Alive and Thrive et al., 2024).

Disaster situations harm health and survival, including through suboptimal infant and young child feeding (IYCF). To protect the health of infants and young children in emergencies, the human rights-based Operational Guidance on Infant Feeding in Emergencies (OG IFE) was published by the Emergency Nutrition Network (ENN) in 1999 and is regularly updated (Interagency Working Group on Infant and Young Child Feeding in Emergencies, 2017). The WHA endorsed the OG IFE in a 2010 WHA Resolution 62.63 (WHO, 2010) and, in 2018, resolved that all Member States implement an emergency plan based on it (WHO, 2018).

For disaster preparedness, the OG IFE recommends sensitizing relevant Infant Feeding in Emergency (IFE) support personnel (including government staff, sector leads, donors, and rapid-response personnel) on psychosocial issues, nutrition screening, and referral pathways to more specialist support. Assessing pre-emergency feeding practices as well as needs and priorities in emergencies is crucial, particularly for addressing the acute challenges faced by vulnerable groups, including children, and for evaluating available resources, such as water.

Various challenges and barriers to breastfeeding in disaster situations in both middle- and high-income countries were highlighted in a recent scoping literature review by Hwang et al. (2021). The biggest challenge identified in establishing optimal infant feeding in emergencies was violations of the WHO International Code of Marketing of Breastmilk Substitutes (BMS), an international health policy framework designed to regulate the marketing of BMS (WHO, 1981; Hwang et al., 2021).

Indonesia is a middle-income country consisting of more than 17,000 islands. In 2023, 3,238 disasters were recorded, and 98,502 people were affected (National Disaster Management Body, 2024). The top three disasters were climate-related: landslides, tornados, and floods (National Disaster Management Body, 2024). Among those affected were many from “vulnerable groups,” defined by the government as infants, children, and pregnant and lactating women, as well as elderly people. In 2021, more than 4 million babies were born in Indonesia (Badan Pusat Statistik, 2022), and the population of infants and young children (aged 0–2 years) was around 13 million (Indonesian Ministry of Health, 2021).

In 2018, the prevalence of exclusive breastfeeding among infants aged up to 6 months in Indonesia was 37.3%, and this varies considerably across different parts of Indonesia (National Institute of Health Research and Development, 2018). This highlights how large the vulnerable group of infants and young children is who are in need of protection in emergency and disaster policy and planning; however, little is known about how effectively government policy in Indonesia protects health and lives by supporting and enabling mothers to breastfeed optimally in such situations. In this study, we aimed to examine the extent to which Indonesia supports and protects breastfeeding during natural disasters and climate-related emergencies

## Method

### Design

We conducted a policy audit of Indonesian regulations on IYCF-E and used content analysis methods to examine how these policies integrated the recommendations of the OG IFE. For inclusion, policies had to containIndonesian government regulations or laws on emergency and disaster responses. We did not include those that did not have specific provisions on infant and young child feeding. Table 1 describes the exclusion and inclusion criteria.

**Table 1.** Exclusion and Inclusion Criteria.

Exclusion Criteria	Inclusion Criteria
Did not mention any information on breastfeeding or infant and young child feeding.	Regulation or law
Mentions breastfeeding mothers as one of the vulnerable groups, but without detailed information regarding the support provided or programs available for them.	Describes the breastfeeding mothers and provides detailed information.
Guidelines published by education institutions.	

### Setting and Relevant Content

Indonesia is a middle-income country in South east Asia. It has five levels of government: national, provincial, city/regency, sub-district and urban suburbs/rural villages (The Australia Indonesia Centre, 2019). The Badan National Penanggulangan Bencana (BNPB) or National Disaster Management Body developed a disaster risk index to map which cities or regencies are disaster-prone in Indonesia (National Disaster Management Body, 2023). The three cities/regencies with the highest risk score are South Halmahera, Southwest Maluku, and Mt Sitoli City, where vulnerable groups represented 44%, 50%, and 43% of the population, respectively (Ministry of Health, 2021). The numbers of births in these provinces in 2022 were 27,182, 35,172, and 25,739, respectively (Ministry of Health, 2021).

Indonesia has adopted the 1989 Ten Steps to Successful Breastfeeding (Ten Steps) into its national regulations (Indonesian Government, 2012), but there is no Baby Friendly Hospital Initiative (BFHI) accreditation program in place. Challenges to the Ten Steps implementation in Indonesia (Flaherman et al., 2018; Pramono et al., 2022) were similar to the international findings (WHO, 2017). Furthermore, the WHO’s International Code of Marking of Breastmilk Substitute (WHO Code) has not been adopted, allowing unethical marketing practices in Indonesia to continue (Hidayana et al., 2017, 2022).

### Sample: Defining the Articles Reviewed

We identified the Indonesian regulations related to natural disasters and climate-related emergency response and management as well as the planning and procedures for the support and protection of breastfeeding mothers during this situation. We found 513 regulations online, but 509 regulations were excluded because they did not specifically mention infant and young child feeding in emergencies.

### Data Collection: The Search Strategy and Process

**Figure 1.**
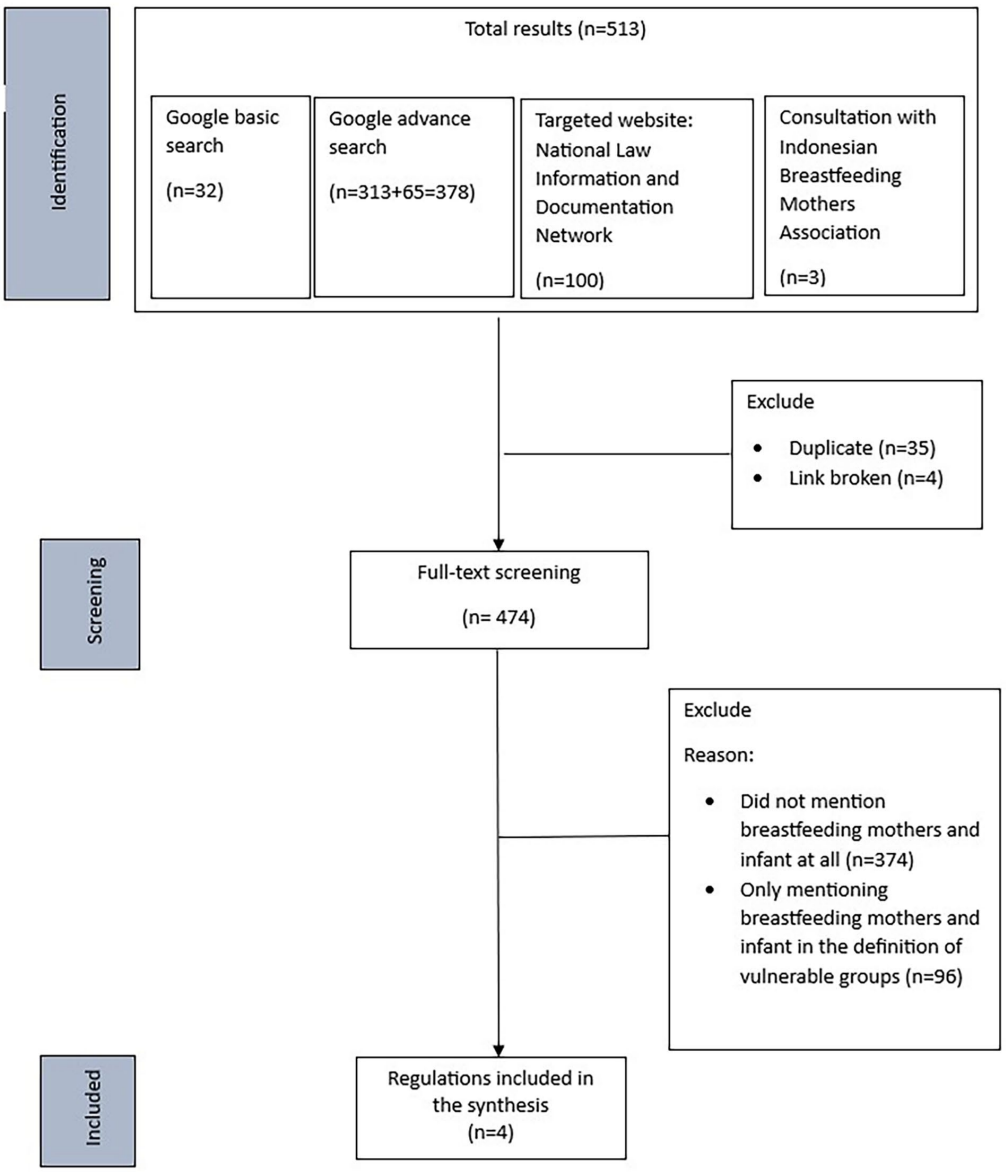
Search Flowchart.

We conducted a grey literature search sequentially in four ways during 2023: (1) Google Basic search; (2) Google advanced search; (3) targeted legal website search; and (4) consultation with the Indonesian Breastfeeding Mothers Association (Asosiasi Ibu Menyusui Indonesia/AIMI) network. The details of the search strategy are described in Table 2 and the search and selection process is shown in Figure 1.

**Table 2.** Summary of Search Strategy.

Number	Search Tool	Search Date	Conducted By	Keywords Used	Results
1	Google Basic	March–April 2023	AH	“Regulation,” “disaster” (in the Indonesian language) AND the name of the province, city, OR region, OR the name of the government organization.	Thirty-three regulations were found, consisting of: -2 regulations at the national level, -23 regulations at the provincial level, -7 regulations at the city/regency level; two of which were instructional letters (IL) -1 Instructional Letter published by authorities at the national level (Health Ministry) but specific to one region.
2	Google Advanced	November 2023	AP, AH, SH	“Regulation,” “disaster” (in the Indonesian language), AND the name of the province, city, or regency.	A total of 313 regulations were found in 157 out of 192 cities and regencies with high-disaster-risk scores (above 144.0). Each high-risk city and regency had regulations regarding disasters, ranging from 1 to 8 regulations.
3	Targeted website of the National Law Information and Documentation Network (www.jdihn.go.id)	December 2023	AP	“Regulation,” and “disaster” (in the Indonesian language).	One hundred regulations were found, consisting of: -69 regulations at the national level, -29 at the provincial level and -2 at the city/regency level.
4	Consultation with the Indonesian Breastfeeding Mothers Association (Asosiasi Ibu Menyusui Indonesia/AIMI) network	January 2024	AH, SH	n/a	Three Instructional Letters were found consisting of: -Instructional Letter of Secretary of Lumajang Regional Government number 441/2941/427.55/2021 about Policy on Milk Formula Donation for Infants and Children Impacted by the Mt Merapi Eruption -Instructional Letter of Cianjur Regional Head of District Health Office number 441.8/10839/Kesmas/2022 about Policy on Milk Formula Donation for Infant and Children Impacted by Earthquake -Instructional Letter of Directorate General of Public Health, Ministry of Health number KK.03.01/V/769/2018 about Policy on Milk Formula Donation for Infant and Children Impacted by the Earthquake and Tsunami in Palu-Donggala, Central Sulawesi.

### Measurement

We manually searched and identified as many relevant policy documents as practicable based on the selection criteria and source. In this title screening stage, regulations addressing disasters but not mentioning breastfeeding mothers or breastfeeding support were excluded. Then, in the full-text screening stage, we excluded regulations that only mentioned breastfeeding mothers as one of the vulnerable groups, but without providing further explanation on the protection or support of infant and young child feeding. AP, AH and SH did the screening and these team members met online via Zoom five times, as well as AP meeting with JS, to discuss the progress of the screening and to resolve any difficulties. We did not limit the year of publication.

### Data Analysis

In the collected documents, the following terms were searched, “breastfeeding,” “breastmilk,” “mother,” and “infant.” All the terms used were in the Indonesian language (“*menyusui*,” “*ibu*,” and “*bayi*”).

We employed qualitative content analysis (Krippendorff, 2019), which, in contrast to quantitative content analysis, is not an automatic process of counting manifest text elements but instead requires in-depth study. Qualitative content analysis can be either inductive or deductive. In this study, we used a deductive approach; the policy documents were analyzed according to predefined themes based on the latest OG IFE (Interagency Working Group on Infant and Young Child Feeding in Emergencies, 2017). These themes are: (1) Endorse or develop policies; (2) Train staff; (3) Coordinate operations; (4) Assess and monitor; (5) Protect, promote and support optimal infant and young child feeding with integrated multi-sector interventions; and (6) Minimize the risks of artificial feeding.

### Trustworthiness

The trustworthiness of the content analysis was evaluated using Lincoln and Guba’s criteria: credibility, transferability, dependability, and confirmability. Credibility was achieved by describing sufficient detail regarding the data analysis process. The multidisciplinary investigators collaborating on this analysis included qualified breastfeeding counselors (AH, SH, and JS), a lactation consultant (AP), and an international IYCF policy expert (JS). On transferability, three of the authors (AP, AH and SH) are Indonesian, and one is an Australian academic researcher (JS) with international policy experience. To demonstrate dependability, all raw data and analysis processes were documented to provide an audit trail. Confirmability was established through multiple team meetings to plan, design, conduct, and discuss the data collection and analysis.

## Results

Although online searches retrieved a total of 513 regulations, only four were included for audit. After review, 509 regulations were excluded because they did not specifically mention infant and young child feeding in emergencies. We analyzed four regulations using the IYCF-E framework (Interagency Working Group on Infant and Young Child Feeding in Emergencies, 2017). These four regulations were for specific locations and disasters, and at a lower level of government (IL). The details of the included regulations are in Table 3.

**Table 3.** Included Regulations and Analysis Compared to the OG IFE.

	Regulation 1^a^	Regulation 2^b^	Regulation 3^c^	Regulation 4^d^		
**Name of Regulation**	Government regulation Number 33, 2012	Instructional Letter of Secretary of Lumajang Regional, 2021	Instructional Letter of Cianjur Regional Head of District Health Office, 2022	Instructional Letter of Directorate General of Public Health, Ministry of Health, 2018		
**Type of Regulation**	Government regulation	Instructional Letter	Instructional Letter	Instructional Letter		
**Level of Government**	National	City/regency	City/regency	City/regency	OG IFE section Addressed^e^	Commentary
**Direct Quotes From Regulations Specific to IYCF-E**	1. Administrator of health care facility is prohibited to give infant milk formula and/or other baby products which can hinder exclusive breastfeeding program; either to mother or her families, excluding those mentioned in Article 15. 2. administrator of health care facility is prohibited to accept and/or promote infant milk formula and/or other baby products which can hinder exclusive breastfeeding program. 3. In case of disaster or emergency, the administrator of health care facility can accept donation of infant milk formula and/or other baby products for humanitarian purposes after receiving approval from head of regional health office.	–	–	–	6.1: Do not donate or accept donations of BMS, other milk products, or feeding equipment (including bottles, teats and breast pumps) in emergencies. Practical Step 5.17: Determine infant formula need through individual-level assessment by a qualified health or nutrition worker trained in breastfeeding and infant feeding issues.	**Points 1 and 2** addressed the OG IFE Practical Step 6.1 **Point 3**, there were no clear explanations or indications for issuing approval from the head of the regional health office. OG IFE Practical Steps 5.17 could be incorporated as the requirements for the approval.
		1. Field Officer should protect, promote, and support mother to maintain breastfeeding.	1. Field officer should protect, promote, and support mothers to maintain breastfeeding.	1. Field officer should protect, promote, and support mothers to maintain breastfeeding.	Practical Step 2 (Train Staff).	**Point 1** (Reg. 2, 3, 4) Addressed OG IFE Practical Step 2 (Train Staff). However, it lacks specific field officer training information for protecting, promoting, and supporting the maintenance of breastfeeding.
		2. Considering water and fuel in state of emergency are in short supply, breastfeeding will be vitally important because it is a primary, safe, and optimum feeding.	2. Considering water and fuel during disaster (emergency) are scarce, breastfeeding becomes very important as a primary, save optimal feeding practice.	–	Practical Step 6.21: Determine availability of fuel, water and equipment for safe household preparation of BMS (cleaning, sterilization, reconstitution).	**Point 2** (Reg. 2 & 3) showed how vital water is to safe breastfeeding and the impact of water shortages. OG IFE mentions importance of water and hygiene sanitation several times, e.g., (Practical Step 6.21) information on access to clean water and sanitation, and social norms on hygiene. Reg. 4 does not have this statement.
		3. All donation in form of breastmilk substitute, bottles, and teats should be located under surveillance and monitoring of Lumajang District Health Office.	3. All donation in form of breast milk substitute, bottle, and teats should be located under surveillance and monitoring of Cianjur District Health Office.	2. All donation in form of breast milk substitute, bottle, and teats should be located under surveillance and monitoring of Cianjur District Health Office.	Practical Step 6.1: Do not donate or accept donations of BMS, other milk products or feeding equipment (including bottles, teats and breast pumps) in emergencies.	**Point 3** (Reg. 2 & 3), and **Point 2** (Reg. 4) partially addressed OG IFE Practical Step 6.1 In Reg. 1, the donation is still enabled but with monitoring and surveillance from the district health office.
		4. The distribution of milk formula should be monitored under health officer along with explanation on preparing and feeding properly and correctly.	4. The distribution of milk formula will be conducted under health officer with explanation of preparing and feeding properly and correctly.	3. The distribution of milk formula will be conducted under health officer with explanation of preparing and feeding properly and correctly.	Practical Step 6.25: Do not use general or blanket distributions as a platform to supply BMS. Practical Step 6.27: When BMS are distributed, ensure there is adequate breastfeeding counseling and support for breastfeeding mothers.	**Point 4** (Reg. 2 & 3), **Point 3** (Reg. 4) addressed IG OFE Practical Step 6.25, which further states that dried and liquid milk should not be distributed alone in general or widespread distributions, as they might be used as breast milk substitutes, which could pose risks to both breastfed and non-breastfed infants. This is where the role of the district health office should be to explain preparing and feeding properly and correctly. Furthermore, the Practical Step 6.27 recommended providing sufficient breastfeeding counseling and support for mothers when distributing BMS and offering valuable items to breastfeeding mothers, such as food or hygiene products. We believe this important step should also be addressed in the regulation.
		5. The distribution of milk formula should be in company with bottled drinking water due to lack of clean water and electricity.	5. The distribution of milk formula should be in company with bottled drinking water due to lack of clean water and electricity.	4. The distribution of milk formula should be in company with bottled drinking water due to lack of clean water and electricity.	Practical Step 6.21: Determine availability of fuel, water and equipment for safe household preparation of BMS (cleaning, sterilization, reconstitution). Practical Step 6.22: Liaise with WASH provider agencies to secure priority access of families with infants using BMS to WASH services and meet minimum standards.	**Point 5** (Reg. 2 & 3), **Point 4** (Reg. 4) Aimed to reduce the risk of lack of clean water, as recommended in Practical Step 6.21. However, the OG IFE does not mention bottled drinking water, which can also have environmental impact. Instead, in Practical Step 6.22, recommends liaising with WASH provider agencies.
		6. The usage of unclean water in preparing milk formula will increase the risk of diarrhea incident and other infection in infant and toddlers.	6. The usage of unclean water in preparing milk formula will increase the risk of diarrhea incident and other infection in infant and toddlers.	–	Practical Step 5.40: Anticipate and assess the impact of human and animal infectious disease outbreaks on IYCF, such as interrupted access to health and feeding support services; deterioration in household food security and livelihoods; transmission risks via breastfeeding; and maternal illness and death.	**Point 6** (Reg. 2 & 3) indirectly addressed Practical Step 5.40 which recommends to predict and evaluate the impact of infectious disease outbreaks on IYCF, including disrupted services, food security, breastfeeding risks, and maternal health, and take measures to mitigate these risks, consulting the WHO for guidance. Reg. 4 does not have this statement.
		7. Formula feeding practice will be using cup or glass for its ease in cleaning process. Bottle and teats are not suggested for its difficulties in cleaning and easily contaminated.	7. Formula feeding practice will be using cup or glass for its ease in cleaning process. Bottle and teats are not suggested for its difficulties in cleaning and easily contaminated.	5. Formula feeding practice will be using cup or glass for its ease in cleaning process. Bottle and teats are not suggested for its difficulties in cleaning and easily contaminated.	Practical Step 6.23: Discourage use of feeding bottles and teats due to high risk of contamination and difficulty with cleaning. Support use of cups (without spouts) from birth.	**Point 7** (Reg. 2 & 3), or **Point 5** (Reg. 4) addressed Practical Step 6.23. Furthermore, the OG IFE mentioned when a bottle is used it needs to be accompanied by education on safe preparation for bottle feeding.
		8. In case of emergencies, formula feeding can be given to children under 24 months, temporarily until mother is able to relactate. This condition excludes motherless baby, with no breastfeeding since birth and mother had severe sickness.	8. In case of emergencies, formula can be given to children under 24 months, temporarily until mother is able to relactate. This condition excludes motherless baby, with no breastfeeding since birth and mother had severe sickness.	6. Formula can be given to children under 24 months, temporarily until mother is able to relactate. This condition excludes motherless baby, with no breastfeeding since birth and mother had severe sickness.	Practical Step 5.16: BMS requirement may be temporary or longer term. Temporary BMS indications include: during relactation; transition from mixed feeding to exclusive breastfeeding; short-term separation of infant and mother; short-term waiting period until wet nurse or donor human milk is available.	**Point 8** (Reg. 2 & 3) **Point 6** (Reg. 4) addressed Practical Step 5.16 which stated that BMS needs can be temporary (e.g., relactation, short-term separation) or long-term (e.g., pre-crisis non-breastfeeding, HIV, orphaned, maternal absence or illness, specific medical conditions, or maternal choice).
		9.Donated formula can be consumed as the main ingredient for nutrient-dense dishes such as traditional snacks or pudding, especially distributed to toddlers, pregnant mothers, and elder people.	9. Donated formula can be consumed as the main ingredient for nutrient dense dishes such as traditional snacks or pudding, especially distributed to toddlers, pregnant mothers, and elder people.	7. Donated formula can be consumed as the main ingredient for nutrient dense dishes such as traditional snacks or pudding, especially distributed to toddlers, pregnant mothers, and elder people.	Practical Step 6.25: Do not use general or blanket distributions as a platform to supply BMS. Dried milk products and liquid milk should not be distributed as a single commodity in general or blanket distributions as they may be used as a BMS, exposing both breastfed and non-breastfed infants to risks. Dried milk products can be pre-mixed with a milled staple food for distribution to use as a complementary food in children over 6 months of age. Where milk powder is commonly used or widely available in a population, recommend and provide practical guidance to incorporate into cooked family meals and advise against use as a BMS.	**Point 9** (Reg. 2 & 3) **Point 7** (Reg. 4) addressed Practical Step 6.25 which stated powdered milk can be combined with a ground staple food for distribution as a supplementary food for children older than 6 months.
		10. Donated milk formula must have expiration date minimum of 1 year from the distribution date”	10. Donated milk formula must have expiration date minimum of 1 year from the distribution date”	8. Donated milk formula must have expiration date minimum of 1 year from the distribution date”	Practical Step 6.17: Supplies should have a 6-month shelf-life from point of delivery.	**Point 10** (Reg. 2 & 3) **Point 8** (Reg. 4) addressed OG IFE Practical Step 6.17 However, in the 2^nd,^ 3^rd^ and 4^th^ regulations a minimum expiration date of 1 year was requred.

Note. BMS = breast milk substitute, Reg = regulation, WASH = water, sanitation, and hygiene, IYCF = infant and young child feeding, OG IFE = Operational Guidance on Infant and Young Child Feeding in Emergencies.

a Regulation 1: Pemberian ASI Eksklusif (Exclusive Breastfeeding), Government Regulation Number 33, 2012 about Exclusive Breastfeeding (Indonesian Government, 2012). (Article 18, page 12-13) https://peraturan.bpk.go.id/Details/5245/pp-no-33-tahun-2012. ^b^Regulation 2: Policy on Milk Formula Donation for Infant and Children Impacted by Mt Merapi Eruption (Government Number 441/2941/427.55/2021, Lumajang Regional Government, 2021) https://anu365-my.sharepoint.com/:b:/g/personal/u6292940_anu_edu_au/EdWh-lcCyvpMpnPKTbUaMxgBFc-OBY6Qq4L5yDs_lFY2DA?e=1clT8t. ^c^Regulation 3: Policy on Milk Formula Donation for Infant and Children Impacted by Earthquake (number 441.8/10839/Kesmas/2022; Cianjur Regional Head of District Health Office, 2022) https://anu365-my.sharepoint.com/:b:/g/personal/u6292940_anu_edu_au/EafBD7-QlM9HniY0IlCTIcABNEEpndDatRvgx3Eb_fxsYA?e=sXYNrT. ^d^Regulation 4: Policy on Milk Formula Donation for Infant and Children Impacted by Earthquake and Tsunami in Palu-Donggala, Central Sulawesi (number KK.03.01/V/769/2018; Directorate General of Public Health, 2018). https://anu365-my.sharepoint.com/:b:/g/personal/u6292940_anu_edu_au/EVPRCRGZGpdKr9p-zhgRrkYBeSbHfU4AiBc8SWoQMPcYBA?e=DaHDYF. ^e^OG–IFE: Interagency Working Group on Infant and Young Child Feeding in Emergencies. (2017). Infant and Young Child Feeding in Emergencies: Operational Guidance for Emergency Relief Staff and Programme Managers. www.ennonline.net/operationalguidance-v3-2017

### Endorse or Develop Policies

The four regulations stated policies on specific issues covered by the OG IFE but lacked explanations on how the government would develop and implement policies for protecting, promoting, and supporting breastfeeding; managing artificial feeding and complementary feeding; and addressing the nutritional needs of pregnant and lactating women. They also mostly did not cover compliance with the WHO Code and relevant World Health Assembly (WHA) Resolutions, preventing and managing BMS donations, or handling infant feeding during public health emergencies and infectious disease outbreaks.

### Train Staff

None of the four regulations included explained the need for staff training on IYCF practices.

### Coordinate Operations

Of the four regulations included, none have descriptions of coordination work on the protection and support of IYCF.

The annexes of the ILs specify other agencies, such as the head of Social Services and the head of communication and digital affairs at the regency level, as the recipients of these letters, showing a coordination structure. However, not all provinces or local governments had published these kinds of regulations.

### Assess and Monitor

No content on the need for prior assessment and monitoring of IYCF practices was found in the four regulations. Specifically, the ILs showed a coordination structure but no specific responsibility for each of these agencies.

### Protect, Promote, and Support Optimal Infant and Young Child Feeding With Integrated Multi-Sector Interventions

The hierarchy of rules and regulations in Indonesia are (1) 1945 Constitution; (2) The Decree of the People’s Consultative Council; (3) Law (Undang-Undang) and Government Regulation in Lieu of Law (Peraturan Pemerintah Pengganti Undang-Undang); (4) Government Regulation; (5) Presidential Regulation; (6) Province Regulation; and (7) Regional/Municipal Regulation (Indonesian Government, 2011). *Government Regulation Number 33, Year 2012 About Exclusive Breastfeeding* is a document specifically about exclusive breastfeeding (Indonesian Government, 2012). As a Government Regulation, it was the highest policy regulating breastfeeding protection, promotion, and support, including during disasters and emergencies. However, it did not mention technical information on how to implement its regulations or whose responsibility it was to train staff to support breastfeeding mothers in the event of an emergency in any one location. Therefore, it needs to be supplemented by other regulations at the provincial and/or city/regency level. These policies would need to articulate how this national policy would be achieved in their settings. The three ILs had attachments listing the authorized institutions and organizations they were targeting, but there were no further descriptions of how the stated goals were to be accomplished.

### Minimize the Risks of Artificial Feeding

At the national level, the Indonesian Government published *Government Regulation Number 33 Year 2012 About Exclusive Breastfeeding* (Indonesian Government, 2012). In Article 18(3) of this document, the administrators of health facilities are allowed to accept infant formula donations for humanitarian purposes in cases of disaster or emergency after receiving approval from the head of the regional health office. Some authorities at the city/regency level can publish an IL, which usually regulates what is done during a single, specific disaster. For example, the IL of the *Secretary of Lumajang Regency Number 441/2941/427.55/2021 Regarding the Policy on Milk Formula Donation for Infant and Young Children Impacted by Mt. Merapi Eruption*.

The OG IFE (Interagency Working Group on Infant and Young Child Feeding in Emergencies, 2017) specifically recommends that governments monitor for WHO Code violations and report breaches to national authorities to minimize the risks of artificial feeding. These measures were not included in any of the four regulations. The national regulation explains only that formula donations are allowed to be received with approval from the local health office. However, the authors of the ILs at the city/regency or regional levels stipulated that all formula, bottle, and teat donations would be under the supervision of the health department at the city or regency level, with formula donation to be accompanied by bottled mineral water and given to babies using a cup.

## Discussion

Governments have agreed to improve planning and preparation for infant and young child feeding. Indonesia is a country prone to disasters and, as in any disaster, mothers, infants, and young children are especially vulnerable. In this review, we accessed Indonesian and English sources to assess Indonesian regulations of IYCF in disaster responses. We found that while national policies exist, they are non-specific, not integrated or comprehensive in scope, and are not systematically implemented at the city/regency level or subdistricts. We found no evidence that staff training on breastfeeding was being carried out in the policies we reviewed. Furthermore, the relation to OG IFE and WHA resolutions was limited; they were at a low level of power in the government system, and were not resourced, communicated, enforced, or integrated with either international or local NGO planning and emergency planning, which is far from a comprehensive or coordinated approach to IYCF for disaster situations.

The existing regulations did not align policies with the international recommendations in the OG-IFE. For example, the international policy on formula donation states the need to avoid donating or accepting breast milk substitutes, other milk products, or feeding equipment due to various risks and challenges, and ensure donor human milk is only sent if needed and part of a coordinated intervention, while clearly communicating policies and informing potential donors about appropriate alternatives. Contrary to this, our assessment of the policies we reviewed had no measures in place to prevent uncontrolled distribution to non-target groups (breastfed infants). This creates the possibility for formula companies to use disasters to create new markets among populations that previously breastfed. In Yogyakarta, this has contributed concerningly to higher rates of child illness in communities after previous earthquake disasters in Indonesia (Hipgrave et al., 2012).

Our search of the research literature indicates that there is a lack of Indonesian studies regarding breastfeeding support in emergency settings, which suggests a low level of awareness of this critical nutrition and health issue among Indonesian scholars and policy decision-makers.

In practice, most IYCF support during emergencies in Indonesia is accomplished through collaboration with non-governmental organizations. For example, the Indonesian Breastfeeding Mothers Association (Asosiasi Ibu Menyusui Indonesia/AIMI) and the United Nations Children’s Fund (UNICEF) Indonesia opened and operated a public kitchen to prepare suitable complementary foods for children under 1 year of age after Situ Gintung dam burst in Tangerang, West Java, in 2009 (Asosiasi Ibu Menyusui Indonesia, 2009). These organizations also supported mothers to re-lactate or maintain breastfeeding after the disaster. Most AIMI volunteers were breastfeeding counselors who had attended the WHO/UNICEF’s 40 hours of breastfeeding counseling training. The actual and potential contribution of such local organizations to effective first responses of this kind is an important area for integration with policy at the provincial and/or city/regency level.

Indonesia is not alone in lacking preparedness and planning for safe and appropriate IYCF in emergencies. It is also evident globally, including in European countries (Iellamo et al., 2024; Zakarija-Grković et al., 2020) and Australia (Gribble et al., 2019). Lack of breastfeeding awareness from the emergency-response officers, and inadequate facilities for mothers to breastfeed and care for IYC at evacuation camps, have been highlighted in several studies (Giusti et al., 2022; K. Gribble et al., 2023; MirMohamadaliIe et al., 2019).

The general regulations on IYCF-E that have been published by the three regions that have the highest disaster-risk score (South Halmahera, Southwest Maluku, and Mt Sitoli City) mentioned breastfeeding mothers as part of vulnerable groups, but were not included in our review because they did not provide any action plan to support breastfeeding. This reveals the need to allocate specific funding for improvements to IYCF-E planning and preparations at the sub-national level, as such jurisdictions typically lack adequate funding for activities needed to support breastfeeding.

Some mothers who did not breastfeed before natural disasters, or mothers who stopped breastfeeding due to the disaster, can be encouraged to start breastfeeding or relactate. Azad et al. (2019), found that wet nursing, which refers to the possibility for a surrogate other than an infant’s mother to breastfeed a child, might be feasible in some situations through intensive counseling as an alternative to the formula for infant nutrition in an emergency setting. Smith and Iellamo (2020) reviewed the regulations affecting wet nursing and donor human milk sharing in emergencies in 25 countries but found no guidance to support emergency responders. K. D. Gribble et al. (2023) recommended that to help infants and young children survive during situations like the 2019–2020 Australian bushfire disaster emergency, the primary focus needs to be on supporting their caregivers.

A major challenge remains in Indonesia, where BMS donation and distribution are not adequately controlled during emergency situations in many areas. There were only three ILs that covered this topic area, and it is possible that the implementation of the ILs in the field might not be effective. Between the scarcity of regional policy and the weakness of the strategy suggested in the ILs, there is likely to be a continued risk of uncontrolled distribution of formula as the policies might not be enforceable or effective in practice, even though, according to the policy review, there were some localized (IL) stipulations that controlled the distribution in specific emergencies. Although the negative impact of BMS donation during emergencies has been shown in many research studies (Dall’Oglio et al., 2020; Ratnayake Mudiyanselage et al., 2022; Theurich et al., 2019), including one in an Indonesian setting (Hipgrave et al., 2012), many countries lack suitable regulations regarding BMS donation during emergencies (Gupta et al., 2019).

### Limitations

The key limitation of our study was that not all regulations, especially at the city/regency level, are available online. Therefore, we had to use individual outreach by contacting the Head of Advocacy and Law at regions where disasters have occurred, to capture these regulations. The results of this review were very local, and may not apply to other LMICs

## Conclusions

Our audit of relevant regulations demonstrated that, despite experiencing many disasters, Indonesia still lacks awareness and comprehensive planning for breastfeeding support and protection during emergencies aligned with WHA Resolutions 63.23 and 71.9 for ensuring evidence-based and appropriate infant and young child feeding during emergencies. Improvement in emergency response planning and preparations, including integration and coordination with international agencies and local breastfeeding support organizations, and providing a safe and baby-friendly environment in evacuation facilities in line with OG IFE, is urgently needed. Measures to prevent uncontrolled formula distribution, in particular, are evidence-based measures that address protecting the health and well-being of children and their mothers in disasters and emergencies in Indonesia.
